# Deciding to Disclose a Mental Health Condition in Male Dominated Workplaces; A Focus-Group Study

**DOI:** 10.3389/fpsyt.2018.00684

**Published:** 2018-12-19

**Authors:** Elizabeth Stratton, Rochelle Einboden, Rose Ryan, Isabella Choi, Samuel B. Harvey, Nicholas Glozier

**Affiliations:** ^1^Brain and Mind Centre, Sydney Medical School, University of Sydney, Sydney, NSW, Australia; ^2^School of Psychiatry, University of Sydney, Sydney, NSW, Australia; ^3^School of Psychiatry, University of New South Wales, Sydney, NSW, Australia; ^4^Black Dog Institute, Sydney, NSW, Australia; ^5^St George Hospital, Sydney, NSW, Australia

**Keywords:** male-dominated, disclosure, mental illness, employee, decision aid tool

## Abstract

**Objectives:** Deciding to disclose a mental illness in the workplace requires thoughtful informed decision making. Decision aids are increasingly used to help people make complex decisions, but need to incorporate relevant factors for the context. This study aimed to identify factors and processes that influence decision making about such disclosure to inform the development of a disclosure decision aid tool for employees in male dominated industries.

**Methods:** We invited 15 partner organisations in male dominated industries to facilitate the recruitment of employees who either had disclosed a mental health condition in their workplace; or occupied a position to whom employees disclosed to focus groups addressing the aims.

**Results:** The majority of the organisations had explicit policies that employees must disclose and so were unable to be seen countenancing non-disclosure as an option. Two focus groups were conducted (*n* = 13) with mainly male (62%), full-time employees (85%), and both disclosed (46%) and authority (54%) groups. Six themes, all barriers, were identified as influencing decision making processes: knowledge about symptoms, and self-discrimination (internal), stigma and discrimination by others, limited managerial support, dissatisfaction with services, and/or a risk of job or financial loss (external).

**Conclusion:** Decisions to disclose mental health conditions, even by those who had done so, appear driven entirely by consideration of negative aspects. This suggests that anti-discrimination policy, legislation, awareness campaigns, and manager training have yet to change negative perceptions, and that any decision aid tool needs to incorporate counterfactual positive aspects that appear not to be an important consideration in such male dominated workplaces. There is a disconnect between organisational policies favouring disclosure and employees favouring non-disclosure that has caused tension within the organisational culture. Decision aid tools may assist employees with an active disclosure without waiting for an event to occur, giving the control of the decision back to the employee.

## Introduction

Despite the high prevalence, mental illness in the workplace is commonly misunderstood and under supported (1), even though employment plays an important role in maintaining psychological health, and for men, paid work is associated with increased levels of personal growth (2).

In Australia, male dominated industries are defined as workplaces where more than 70% of the working population are males (3) and in Australia male dominated industries tend to have higher than average rates of common mental disorders with the mental disorder rates in agriculture, construction, mining, and utilities ranging between 20 and 24% (4).

When considering workplace compensation claims made in Australia between 2008 and 2011 the highest risk male occupations for metal stress are transport officers, police officers and ambulance officers/paramedics (5).

People in employment often face complexities in managing mental illness as a result of discrimination or stigma. This is particularly true when (re)entering the workforce after being acutely ill or trying to continue working despite an illness, with almost 70% of workers reporting anticipated discrimination when finding work or trying to manage their symptoms at work (6).

Discrimination, both anticipated and experienced, is the most frequently identified barrier to disclosure of mental health conditions in the workplace (7). Recent studies have shown that employers were less likely to hire someone who discloses their mental illness during recruitment, even more so than people with permanent physical disabilities (8, 9). In a workplace context, stigma and discrimination are important factors that hinder those with a mental health condition in seeking employment, staying employed, or gaining a promotion (8, 10). Discrimination and bullying can vary between industries with male dominated industries reporting experiences of discrimination associated with reduced mental health [(11), paper under submission] and female dominated industries more often reporting bullying (12).

For individuals making decisions around their disclosure important factors to consider are the organisational policies around disclosure and if these policies align with that of the individual. Organisational policies promote disclosure and managers report preferring disclosure, even though they acknowledge that it would be a significant risk to employ those with a disclosed mental illness (13). A recent European longitudinal study on attitudes towards disclosure in employers showed that 42% of employers agreed that mental illness policy was set up primarily to protect the organisation from litigation (14). However, fear of perceived public stigma has lead employees to default to a position of non-disclosure (15).

Many countries have enacted legislation to tackle such discrimination, usually framed in a disability context. The Australia Disability Discrimination Act 1992 (Cth) (16) defines “discrimination” broadly, and includes direct and indirect discrimination. The Act makes it unlawful to discriminate against, harass or victimise people with disabilities, including those with mental health conditions. It also makes it clear that an employer's reluctance to hire, or failure to make reasonable adjustments for a worker with a mental health condition are discriminationatory practices. Organisational practices and commonly accepted behaviours occur and remain unchallenged, despite falling within the legal definition of indirect discrimination.

In order to address gaps in knowledge regarding mental health in the workplace, a number of workplace manager interventions have been implemented with an emphasis on awareness training and skills development. A recent meta-analysis indicated that mental health training appears to increase managers' understanding of issues, their role and responsibilities, attitudes, and importantly, behaviours when addressing mental health concerns (17). Workplace anti-stigma interventions follow a similar process to manager training by targeting knowledge, attitudes and behaviours. A recent systematic review reports that workplace anti-stigma interventions can be particularly effective in changing employees' knowledge of mental disorders, as well as helping change behaviour of colleagues in the workplace (18).

Despite interventions aimed at managers and colleagues, employees are still reluctant to disclose. A recent Australian survey showed that 41% of those who had taken unplanned leave for their mental illness did not disclose the true reason to their employer, and only 26% disclosed their mental illness (19). Research on disclosure of mental health conditions in a workplace setting has typically focused on those with more severe mental illness (20) with interventions focusing on those that are unemployed to help facilitate return to work (21). However, little attention has been paid to helping employees make the decision to disclose mental health issues in the workplace.

For people currently in employment, deciding to disclose a mental illness is often complex, and requires thoughtful decision making to ensure personal, legal and employment risks are best managed. Decision aid tools offer a way to support people with these complexities. They can encourage thoughtful deliberation in making specific and deliberate choices amongst two or more options, and have commonly been used in decision making regarding medical treatment options (22). The recent success of the CORAL study (21), which used a paper-based decision aid tool for people with a severe mental illness in secondary care services seeking employment showed that, in this setting, the decision aid had the capacity to reduce decisional conflict. Those using the tool had an increased likelihood of obtaining employment regardless of the decision made, indicating the importance of making, and being comfortable with an informed decision.

As part of the future development of a web-based decision aid tool to support deliberations of disclosure of mental health conditions in the workplace we aim to explore factors currently influencing decision making about disclosure with two key groups, employees who have disclosed and supervisors involved in the sequelae to disclosure.

## Methods

### Design

This qualitative focus group study aimed to inform the design of a tool to help employees in male dominated industries *decide* whether to disclose or not, and describe factors that influence the decision process.

We aimed to recruit participants that: (a) have a mental health condition and have disclosed; or (b) occupy a position of authority, i.e., supervisors who's employees with a mental health condition have disclosed to. This participant selection strategy offered insights into perceptions among those who had experience in decisions regarding disclosure, as well as those who managed disclosures. This qualitative strategy allowed access to a variety of perspectives and experiences, and maximised our ability to identify challenges in decision making and develop an effective, appropriate intervention by being able to open dialogue and expand on responses when clarification is needed.

### Sample/Recruitment

As a part of a large Australian based research collaboration (well@work) 15 industry partners involved were invited to participate. Participants were recruited from a First Responder Association and a transport company in New South Wales, Australia. Twenty-Three employees and supervisors were invited through an email sent out via their workplace administration team. Interested participants were directed by the administration team at each site to attend the focus groups. The research team had no contact with participants until the day the focus groups were conducted. Ethical approval was obtained from the University of Sydney.

### Procedures

After obtaining informed consent, each participant was asked about their role, and if they wished to disclose their mental illness. Those who receive disclosures were considered the “authority group” and those who have disclosed were considered the “disclosure group.” The focus groups were conducted at organisational sites and lasted between 77 and 93 min.

### Data Collection

The focus group facilitator (ES) was trained by (RR) an experienced workplace focus group researcher. The facilitator (ES) and the principal investigator (NG) constructed the focus group discussion guide. The questions were designed to assess the process and content of the web-based decision aid tool, and identify factors influencing and consequences to decision making about disclosure. A semi-structured approach was adapted to ensure that the facilitator could follow-up on any important remarks or seek clarification of understanding. Focus groups were audio recorded, transcribed verbatim and accuracy was verified. The transcriptions were de-identified and given a participant identification number as a pseudonym to protect anonymity. Only the main researcher ES had access to the participants identification. After the focus group, the facilitator and trainer met to review the techniques used in facilitation. After both of the focus groups the facilitator met with the principal investigator to debrief. Transcripts were analysed as soon as possible after each interview. After two groups, participants were repeating similar ideas about the design of the tool, prompting a decision that saturation had been reached.

### Data Analysis

Two authors (ES & RE) independently coded the typed transcriptions of the focus groups. NVivo 11 was used to organise the data. The investigators used the Framework Analysis Method (23) to analyse the data (24). This method allows for the structured identification of commonalities and differences in qualitative data, and helps to draw descriptive and explanatory conclusions clustered around common themes. It is especially useful when multiple researchers are coding the same dataset, by providing an open, critical and flexible approach to allow for rigorous step-by-step qualitative analysis (25). This method uses five stages, the first stage familiarisation, began when investigators started transcribing the data. A thematic framework, was then identified by two investigators and coded each transcript separately. The coding involved an iterative process as the codes were refined, while discrepancies were resolved by a third investigator (NG). Next, key themes and direct quotes were indexed to demonstrate the richness of the themes. Subthemes within these key themes provided an in-depth approach to identifying the factors influencing decision making. In the fourth stage, themes were charted (see **Table 2**) as to easily identify the data and trace back to its original source. Each theme was described to reflect key characteristics of the decision making process.

## Results

### Participants

Of the 15 industry collaboration partners invited, only two of the industries would consider facilitating a tool that promotes both disclosure and non-disclosure. All of those who gave a reason stated that they had explicit employment and human resource policies that employees must disclose mental illness. These included both private and non-government organisations.

The two focus group we were able to conduct consisted of 13 participants (57% response rate), employed within the two organisations. The majority were from a Transport Company (69%), male (62%), and had full-time employment (85%) (Table 1). Six employees had experience disclosing their own mental health condition (employee 1, 2, 3, 4, 5, 6) and seven had a supervisory role and had been disclosed to (supervisor 1, 2, 3, 4, 5, 6, 7).

**Table 1:** Demographic Information for Participants.

**Category**	***n***	**%**
**WORKSITE**		
First Responder Association	4	30.8
Transport company	9	69.2
**GENDER**		
Male	8	61.5
Female	5	38.5
**EMPLOYMENT STATUS**		
Part-time	2	15.4
Full-time	11	84.6
**CATEGORY**		
Disclosure	6	46.2
Authority	7	53.8

### Decision Making Influences

Participants reported factors that influenced decision making about disclosing a mental health condition at work. Six key themes emerged, each with several subthemes, including: internal factors (lack of knowledge about symptoms of mental illnesses, self-discrimination) and external factors (stigma and discrimination by others, a lack of managerial support, dissatisfaction and distrust of the current services and a risk of job or financial loss) (Table 2).

**Table 2:** Identified themes of factors that influence decision making in employees with a mental health condition.

**Theme**	**Sub theme**	**Quotes**
Lack of knowledge about mental illness or symptoms	Not knowing if they have a mental illness	Employee 3: “*I think also not knowing if you're just stressed or if you do actually have a mental illness is a big thing, we don't ever get screened or get any mental health assessments at work”* Employee 2: “*you may feel like you're dealing with a depression type situation but you might actually just need to look at controlling your stress”* Employee 6: “*we need some kind of screening measure at work to see our current mental health state”*
Stigma & discrimination by others	Experienced;	
	No reasonable adjustments	Supervisor 1: “*put on restricted duties and basically jobless”* Supervisor 6: “*all bosses need to do is know and comply with the policy in the work health and safety. So as long as there's a reasonable policy in place and they comply with it that's all they do”*
	Not being taken seriously	Employee 1: “*they use it just to bag their staff, if I was to tell my boss that I was struggling he would say oh you're struggling blah blah blah, they never say something like, let's go and have a coffee and talk”*
	Anticipated;
	Harm to career prospects	Supervisor 6: “*There's no way that if I was looking to progress in my career would I go to my boss and say I'm struggling”*
	Labeling; - being placed in the “mental health box” - being viewed as weak, unproductive or a liability - workers compensation “compo case”	Supervisor 1: “*It is labeled that once they disclose a mental illness that they are going to be put on restricted duties”* Supervisor 1: “*disclosure tends to have a negative connotation”* Employee 1: “*you don't necessarily want it to be the boss you know, if you go and tell them you're not well they'll think you're a dickhead mate I don't want to talk to you”* employee 5: “*I think there's a stigma to put everyone in the same basket”* Employee 6: “*so they aren't seen as a “mental health case””* Employee 3*: “I think that often people are afraid to talk about it because they are going to get put in a basket”* Employee 2: “*when you have a pretty severe workload and no resources and can't handle your work they'll think you're weak”* Supervisor 6: “*being a leader I wouldn't have disclosed anyway because it's very much seen as a weakness so yeah I would never have disclosed”* Supervisor 2: “*as soon as you disclose you're a workers compensation case”*
	Worry about what others will think	Supervisor 1: “*it is something that our members run for the hills for, they're too worried about what others will think”* Employee 6: “*I worry about the impact or the stigma that does get raised when you confirm or you admit to it.”*
Self-discrimination	Worry of being a burden	Employee 6: “*Particularly because my new team now know but obviously it doesn't impact on my ability to do my job and I think a lot of people do worry about the impact they might cause”* Supervisor 1: “*they don't tell they just go to a job 1 day and the next day they ring up or the wife rings up and goes he's lying on the floor he won't stop crying and he won't get out of bed. So 0 to 100, the cup fills up and often”*
	Fear of failure	Employee 3: “*I put pressure on myself and, well, if you're anxious then you can't do your job properly”* Supervisor 2: “*We see quite often that people transfer, move and then go off and then there's this theory of you're not letting your friends down that you've work with for the last 10 years because they're new and you don't really know them. And then they will be off and never seen again”*
Managerial Support	Lack of knowledge	Employee 2: “*It's too hard to handle for a lot of supervisors it's just too hard for them to do. And they're not invested enough in it they've got enough on their plate in the sense of other responsibilities so it almost becomes like dynamite. Keep it locked in a safe I don't want to know about it”* Employee 6: “*managers think I don't know what to do to fix the problem”*
	Lack of support or accommodation for illness	Employee 1: “*Supervisors and bosses will walk in and go “nobody needs that EAP shit do they?” Good thank you. And they go tick a box and run out and you go well no one's going to accept it when you call it that EAP shit from the start”* Employee 4: “*I struggled a bit I was going to my boss and saying that I was struggling and you know that I thought things weren't right I didn't feel like I was supported a lot until I got to the point where I broke and I went out”* Employee 6: “*I felt like I was alone in instigating my return to work and I felt like if I was more broken than what I was it would've been an extra strain and pressure on me then I was already dealing with I feel like they didn't really have a good understanding of the whole process and system that I felt needed to be in place”*
	Beliefs creating tension	Supervisor 1: “*why would we reward someone with mental health issues by giving them flexible hours but we can't offer that to those who are coping”*
Poor services and lack of confidentiality	Availability–not knowing where or how to find services	Employee 5: “*I don't know what's available”* Employee 2: “*biggest hurdle is no one wants to go looking for it. Everyone's too busy to look for it”* Supervisor 2: “*there's a huge amount of information on what on what they call “intranet” or on their computer but they don't know where it is”*
	Dissatisfaction;	
	Inadequate services, i.e., no ongoing support, inconsistent care and distrust	Employee 1: “*you should be able to go to that provider throughout your whole EAP, this ridiculous idea of bouncing people around that's a major issue, and doesn't work”* Supervisor 6: “*the biggest problem I find with them is if I go and use EAP I've got my six sessions and once my six sessions are over I've just spent 6 h trusting and building rapport and now I've got to start back from the beginning it's not worth it don't worry about it”* Employee 2: “*there's a lot of dissatisfaction because it's got um, limited use so its capped and also you're not guaranteed to get continuity of service”* Employee 1: “*I could go to three services one on top of the other and I get referred to three different EAP's so there's no consistency”* Supervisor 1: “*the extent of the resources is “go onto the intranet and find the resources and download the PDF” and it all feels a bit static”*
	Anticipated distrust;
	EAP reporting back to employer	Supervisor 2: “*for people offering support you can never promise confidentiality”* Employee 1: “*I'm more likely in the first instance to go external rather than one that the work provides”* Supervisor 1: “*oh Id use EAP but I'm sure what goes back to the employer you never know with those sorts of things”*
	legislative and professional obligations	Employee 2: “*They're more inclined to talk to someone outside or out of that structure who doesn't have to report and who are confidential”* Supervisor 6: “*if it's work related and you put a claim in then you can't stay with that same practitioner you've got to move on into a workers comp space”* Employee 1: “*you may have internal policies or you may be obligated to report this matter or report the way you're feeling currently from the cover or employment point of view”*
Risk of Job or financial loss	Loss of income on use of annual leave or leave without pay	Supervisor 5: “*go and see their own psychologist on their own time and pay for it themselves”* Supervisor 1: “*take annual leave to avoid, or even leave without pay”* Supervisor 4: “*to avoid disclosure you can go off on leave without pay and go and get treated even in hospital treatment on leave without pay using your savings or money”* Employee 2: “*I'd have financial implications if I can't work and it's a big risk”*
	Change in role / No work satisfaction	Employee 1: “*they put you on day shift so you lose your penalty rates, you're losing overtime and people tend to budget on x amount of shifts they're going to be getting. So obviously they go from working out on the field operationally to being behind the counter or something”* Supervisor 2: “*I mean they are supposed to get meaningful work but, they'll put them in charge of something and you know it's just the most boring work they've had and you know they're not using their brain in a way it's just some meaningless task whereas if you engaged in something that required a bit of thought it might take your mind off things, as a distraction”* Supervisor 1: “*may be moved if you tell and it can have travel implications and all sorts of things”*

### Internal Influencing Factors

#### Lack of Knowledge

Some participants, particularly those that had not been previously diagnosed, reported a lack of understanding the severity of their symptoms and not being sure if they even had a mental health condition. Thus, they were unsure if they should seek help. Employee 6 suggested: “*we need some kind of screening measure at work to see our current mental health state.”* A screening tool could assist with assessing their current mental health and confirm if they needed to seek assistance.

#### Self-discrimination

The idea that disclosing a mental health condition would create a burden on others was often cited as consideration in decision making. For instance, Employee 3 stated: “*I put pressure on myself and, well, if you're anxious then you can't do your job properly.”* When participants became overwhelmed with their workload, they reported feelings of failure for not performing to the standard that they expect of themselves. In situations where individuals are feeling pressured and overwhelmed, symptoms of ill-health can reduce decision making capacity and interventions may be useful in assisting the decision-making process in helping to reduce the burden of making a decision.

Another participant reported employees working while they were suffering from a mental health condition and waiting until “*the cup fills up”* (Supervisor 1). Pushing through the symptoms was important so as not to “*let their friends down at work”* (Supervisor 2). However, for some this strategy resulted in becoming so ill that they needed to take unplanned leave.

### External Influencing Factors

#### Stigma and Discrimination by Others

Stigma and discrimination by others was the greatest influencing factor to making decisions of disclosure. Subthemes related to stigma were also identified. The first was “experienced discrimination,” where no reasonable adjustments had been offered and employees disclosures were not taken seriously. Mental health conditions were stigmatised in the workplace, particularly in this setting for first responders. Participants worried about being labelled as a “*mental health case”* (Employee 6), and generalisations being made about their abilities. Across a variety of mental health presentations there was an ongoing worry about being placed “*all in the same basket”* (Employee 5) or being constituted as disabled even when they have their symptoms “*currently under control”* (Employee 2). Participants also reported feelings of those with a mental illness as being constituted as unproductive, a liability or seen as weak, and the implication of such constitutions would result in non-disclosure “*There's no way that if I was looking to progress in my career would I go to my boss and say I'm struggling”* (Supervisor 6). Anticipated discrimination had the largest number of subthemes suggesting it is the most common consideration in decisions to disclose.

#### Lack of Managerial Support

Several participants said they were not supported by their managers during their disclosures because they did not have adequate knowledge of mental illnesses, and already had “*too much on their plates”* (Employee 2) to provide the proper support or empathy needed. These beliefs from both the employees and supervisors led to tension with where their responsibilities lie, often only providing support that they are legally obliged to. Participants conferred that managers did not provide adequate accommodations or support for mental illnesses at work. At times, participants felt belittled or left to fend for themselves when returning to work. One participant, who has been a manager, reported concerns about providing flexibility and support to those who have a mental illness rewards those who were not coping when they cannot provide the same rewards to employees who were coping.

#### Employee Assistance Program (EAP) Services

There was a clear dissatisfaction with, and distrust of, current EAP services. First, participants reported not knowing what was available or where to find services, and feeling overwhelmed about the amount of information to consider when they're already unwell. They questioned the confidentiality of in-person workplace EAP's, and believed EAP support officers were representing both them and the organisation. This mistrust related to legal obligations, particularly in first responders, where staff are required to report their colleagues, if they disclosed mental health conditions. In many ways, confidentiality is limited in their workplace by legislation and professional obligations.

Second, of the participants that had used an EAP previously, most reported the inadequacy of the available programs. Participants identified due to limited sessions, for ongoing treatment, they would need to restart with new EAP officer or be moved off into workers compensation. Employee 1 described: “*I could go to three services one on top of the other and I get referred to three different EAP's so there's no consistency*.” Participants felt they were “*bouncing”* around from one service provider to the next, without gaining any relevant tools for recovery.

#### Risk of Job or Financial Loss

Lastly, participants reported financial and job risks that must be considered when deciding whether or not to disclose. If they disclosed their mental illness, a change in role was so drastically different from their usual one that they were reported as “*boring”* or had “*no meaning”*—Supervisor 2. For first responders, reasonable adjustments were often not available at their workplace and their only option was to relocate. Finally, participants reported using their annual or sick leave and paying for services out of their own pocket to avoid disclosure at work, returning to work only when they felt better. Importantly, they did this even when the onset or exacerbation of the mental illness occurred in or *because* of the workplace environment.

### Positive Experiences of Disclosure

A specific question was asked in both focus groups to identify any positive experiences with, and facilitators of disclosure. Only one responder reported a positive experience, but could not identify how this was enabled. Employee 6: “*I did speak to my manager about it and the triggers. My work has been really good since. We have an EAP service and it was great to experience that service. I think if I didn't have that support those triggers at work would happen a lot more and be more frequent.”*

### Consequences of the Factors Influencing Decision Making, and Non-disclosure

The identified factors influencing decision making about disclosure had a number of behavioural consequences. First, attitudes towards disclosure are seemingly that of, “*it's ok for others to disclose but not for me*” (Supervisor 6). In the authority group, the dissonance between believing that they were supportive but their actions suggesting an unsupportive environment created a culture favouring non-disclosure, resulting in mental health issues rarely being disclosed unless they can no longer be hidden or until seeking help is too late. Even managers themselves reported that they would never disclose if they were considering career progression.

Managers' negative attitudes towards those that report a mental illness at work often lead to a lack of preparation to support the mental health of their employees despite employees often becoming ill or symptoms worsening on duty. Managers responses to mental illnesses at work become paternalistic and protective and the organisations' responsibilities, doing only what is required by law or internal policies and procedures. These behaviours point to a context of little flexibility and support for workers in general creating tensions with a belief that providing flexibility and support “*rewards*” those that are not coping because of their mental illness. Managers felt torn that they cannot provide the same accommodations for those that are coping well with work demands “*why would we reward someone with mental health issues by giving them flexible hours but we can't offer that to those who are coping*” (Supervisor 1).

This managerial discrimination has led to the practice of “reasonable adjustments” becoming simply restricted duties that have a lack of autonomy, are mundane and are not challenging, resulting in poor job satisfaction, poor job security, negative financial implications, isolation and or dislocation, a decreased self-worth in a work context, lack of opportunity for advancement. Employees who have disclosed are left feeling that they need to work harder than others to prove one's worth “*they are supposed to get meaningful work but, they'll be put in charge of something and you know it's just the most boring work”* (Supervisor 2).

Participants reported some consequences of non-disclosure, if an employee does not disclose their mental illness, and there is an incident in their workplace that places themselves, a colleague or a member of the public in harm's way they or their organisation may face legal action. Particularly if they are taking medications for their mental illness that have not been disclosed and may be present in a toxicology report that may have impaired their judgement during the incident. It was also reported that workers may not take their medications that were prescribed in order to avoid disclosure.

Employee 1: “*If someone was involved in a critical incident or something the first thing they're asked is if they take any medications, and if they start listing down different psychological medications and haven't said anything before people may say they were impaired, or affected by it and they shouldn't have been working.”*

Employee 2: “*people just don't take their medications that are prescribed to them because if it's on the banned list or will show up in a tox report you're forced to disclose it. But if you don't take it then you don't have to disclose it.”*

## Discussion

Despite only 25% of those with a mental health condition disclosing their illness at work (19), few studies have investigated the factors which influence decision-making around disclosure. This present study explores these factors among first responders and a transport organisation who either have previously disclosed or were responders to such decision processes. This study identified six key themes, all negative, of internal and external factors influencing and consequences of decision making regarding disclosure. When considering all factors together these male dominated workplaces are characterised by a culture of fear around disclosure. Managers appear underprepared to deal with the impacts of disclosure, and there is a lack of organisational systems in place to deal with mental health issues amongst employees.

The prevalence of explicit disclosure policies led to the majority of our partners declining to be involved in research that consider an option of non-disclosure. A recent qualitative study in Canada suggested that organisational policies should not focus on a stance of disclosure as the only option, the focus should be placed on creating an environment where employees feel comfortable and safe to disclose (15).

This study, similarly to Toth and Dewa (15), identified that employees start from a default position of non-disclosure, and identified that employees only move to a position of disclosure once they feel as though an event occurs such as “their cup fills up.” This dissonance between organisational policies taking a hard stance on disclosure and employees defaulting to the other option of non-disclosure has created tension and created a culture of fear around disclosure.

What was striking was that despite the researchers adopting a neutral approach and asking a specific question to prompt negative or positive experiences to whether disclosure was desirable or not, only one person considered the potential positive aspects of doing so. The single incident of a positive experience in disclosing mental illness suggests a failure of the extensive government initiatives aimed at demonstrating the possibilities of workplace accommodations, and shows dissonance between employers preferring prospective employees to disclose at the outset (26) even though they may not be willing or have the capacity to provide support or adjustments, or even be willing to hire those with a mental illness in the first place (27). Decision aids need to specifically incorporate these factors into the considerations to balance the entrenched barriers. The provision of training for managers and colleagues is not enough to address the barriers, as low rates suggest that many of those living with a mental illness are still not disclosing (19).

Self-stigma and internalisation of discrimination reduces the capacity to make decisions (28). Anticipating discrimination, worry about being a burden, being judged as weak, or being denied a promotion also influences decision making about disclosure supporting previous research that suggests those that have a mental illness often decline to seek help to avoid being stigmatised (29). Poor knowledge of potential mental illness, particularly discriminating early symptoms from daily stress (30) would be present in those not yet at a stage of decision making who may be unaware of options available to prevent deterioration, which might require disclosure. A recent study found that better knowledge, and a positive self-attitude can predict stronger intentions to disclose (31).

A number of external factors to decisions about disclosure were also identified. Some participants had experienced discrimination by others when disclosing in the past; particularly common were lack of support or preparedness of managers to provide reasonable work adjustments after disclosures. Consequences of this included a negative culture around mental illness in the workplace, which, as shown in other studies, seemingly reduces the motivation for employees to disclose (32, 33). Which we know may be detrimental in male dominated industries as men are more likely to disclose than women when there is a supportive environment (34).

Secondly, some employees anticipated discrimination or worried about being seen as weak or incompetent. This anticipated reaction is based on experience and substantiated by research, with previous studies showing that employers view people with mental illness as lacking competence to meet work demands (27, 35).

Both of these first two themes identified could have serious impacts on male dominated industries in particular, as two recent systematic reviews examining the characteristics and risk factors that are associated with common mental disorders in male dominated industries identified that an unsupportive environment, job demands and job overload are associated with psychological distress and are risk factors for depression and anxiety (36, 37).

In contrast to previous research (38), this study identified a clear dissatisfaction and distrust with current EAP services. Participants reported not knowing what was available, found it difficult to find what they needed when they were feeling unwell and overburdened. The inadequacy of the available EAP programs were also reported with complaints about session lengths, inconsistency of care and not gaining any relevant tools for recovery. Confidentiality was another factor identified that influenced decision making, participants had a general belief that EAP officers report details from the sessions to their employers. Confidentiality concerns with EAP services have been previously documented (39), however, this study shows how a lack of anticipated confidentiality can lead to decisional conflict around disclosure. Confidentiality may be limited within colleagues as some legislation and professional obligations require colleagues to report to management if someone has disclosed to them, this may be an issue specific to the Australian first responders group as they have particular safety concerns related to their professional responsibilities.

The final factors that influence decision making identified were financial implications and risk of job loss or role change. A number of participants both from the authority and disclosure groups reported either personal or second hand accounts of seeking help outside of the workplace to avoid disclosure and its consequences, often using up their holiday and sick leave and accruing personal expenses whether the illness originated or was exasperated in the workplace. Other participants also reported worry over the work that will be available to them; work via reasonable adjustments are either mundane and require relocation to have reasonable work available that has been previously shown to increase the prevalence of isolation and reduce communication and team relationships (40).

### Strengths and Limitations

The findings of this study were unique to this particular population and the analysis has been undertaken with the aim to develop a decision making support aid. The theoretical perspectives are oriented towards supporting individuals deciding whether or not to disclose a mental health issue or managing a disclosure, and achieving equity for persons with mental health issues in the workplace. Second, the participants in the focus groups were people who volunteered to participate in a qualitative research, from a group that already have experience with disclosing a mental illness at work. Therefore, this group was likely to be highly motivated to participate in research and may have been different from those who did not wish to share their views, they may have less self-stigma and be able to hold back less with their responses as they had already disclosed. Lastly, this sample may not be representative or generalised to other occupations or industries as participants were recruited from two industries with specific needs, in particular the first responders had unique challenges given legislative requirements and obligations which are specific to only emergency/medical services (41).

## Conclusion

In male dominated workplaces decisions to disclose mental ill-health, even by those who had done so or who were influential for consequences, appeared driven entirely by consideration of barriers to disclosure. This suggests that anti-discrimination policy, legislation, awareness campaigns, celebrity stories, and manager training have yet to change perceptions that disclosure is negative, and have not been able to shift the culture of stigma that looms over mental health issues. There seems to be a clear disconnect between organisational policies favouring disclosure and employees favouring non-disclosure that has caused tension within the organisational culture and a fear around disclosure. Further attention is needed to encourage a more positive and productive approach in supporting employees in the workplace. Any decision aid tool needs to incorporate counterfactual and potentially positive aspects that are not yet widely considered in decisions about disclosing mental health issues in the workplace. Such content could begin to shift cultures of stigma, as well as support people to be properly informed and feel empowered and that they are making the correct decision for their situation.

## Author Contributions

ES, IC, SH, and NG contributed to the design and conduct of the study. RR and ES facilitated the focus groups. RE and ES coded the data. ES, RE, and NG had full access to all of the data and completed the analysis. All authors reviewed manuscript drafts and approved the final version.

### Conflict of Interest Statement

The authors declare that the research was conducted in the absence of any commercial or financial relationships that could be construed as a potential conflict of interest.
